# Survival outcome and predictors of WHO grade 2 and 3 insular gliomas: A classification based on the tumor spread

**DOI:** 10.1002/cam4.7377

**Published:** 2024-06-08

**Authors:** Bowen Xue, Zonggang Hou, Zhenghai Deng, Shengjun Sun, Chuanhao Zhang, Yuesong Pan, Yazhuo Zhang, Zhenye Li, Jian Xie

**Affiliations:** ^1^ Department of Neurosurgery Beijing Tiantan Hospital, Capital Medical University Beijing China; ^2^ China National Clinical Research Center for Neurological Diseases Beijing China; ^3^ Department of Radiology Beijing Tiantan Hospital, Capital Medical University Beijing China; ^4^ Beijing Neurosurgical Institute, Capital Medical University Beijing China

**Keywords:** classification, insular glioma, limbic system, oncology, paralimbic system, survival analysis

## Abstract

**Objective:**

The study aimed to identify if clinical features and survival outcomes of insular glioma patients are associated with our classification based on the tumor spread.

**Methods:**

Our study included 283 consecutive patients diagnosed with histological grade 2 and 3 insular gliomas. A new classification was proposed, and tumors restricted to the paralimbic system were defined as type 1. When tumors invaded the limbic system (referred to as the hippocampus and its surrounding structures in this study) simultaneously, they were defined as type 2. Tumors with additional internal capsule involvement were defined as type 3.

**Results:**

Tumors defined as type 3 had a higher age at diagnosis (*p* = 0.002) and a higher preoperative volume (*p* < 0.001). Furthermore, type 3 was more likely to be diagnosed as *IDH* wild type (*p* < 0.001), with a higher rate of Ki‐67 index (*p* = 0.015) and a lower rate of gross total resection (*p* < 0.001). Type 1 had a slower tumor growth rate than type 2 (mean 3.3%/month vs. 19.8%/month; *p* < 0.001). Multivariate Cox regression analysis revealed the extent of resection (HR 0.259, *p* = 0.004), IDH status (HR 3.694, *p* = 0.012), and tumor spread type (HR = 1.874, *p* = 0.012) as independent predictors of overall survival (OS). Tumor grade (HR 2.609, *p* = 0.008), the extent of resection (HR 0.488, *p* = 0.038), IDH status (HR 2.225, *p* = 0.025), and tumor spread type (HR 1.531, *p* = 0.038) were significant in predicting progression‐free survival (PFS).

**Conclusion:**

The current study proposes a classification of the insular glioma according to the tumor spread. It indicates that the tumors defined as type 1 have a relatively better nature and biological characteristics, and those defined as type 3 can be more aggressive and refractory. Besides its predictive value for prognosis, the classification has potential value in formulating surgical strategies for patients with insular gliomas.

## 1 INTRODUCTION

The insula is one of the most complex structures in the brain and performs various significant functions.^1^, ^2^, ^3^, ^4^ Despite its slow progression, insular gliomas tend to migrate to adjacent brain regions, plaguing patients and neurosurgeons. Anatomical and functional features made insular glioma an intractable problem for many years. In 1992, Yasargil proposed a study to describe relevant surgical approaches and techniques, identifying the availability of resection for insular gliomas.^5^

The insula cortex is one of the last regions to develop with the frontal lobe. Serving as a relay between the neocortex and allocortex, it emerges from the mesocortex. It has complex connections with the limbic system via the entorhinal cortex and the uncinate fasciculus.^6^, ^7^ Hence, we referred to the insula as the paralimbic system. Besides the insula, the orbitofrontal cortex and temporal pole are also components of the paralimbic system, and the prepiriform cortex trifurcates the three limbs of the paralimbic system.^8^, ^9^ The above anatomical characteristics are inseparable from the growth patterns of insular gliomas. Hence, it is not rare that insular gliomas affect surrounding structures. Based on the study suggested by Yasargil, many excellent neurosurgeons have studied the growth patterns of insular gliomas. Duffau presented two studies on the fasciculus and paralimbic system separately, indicating different invasive pathways and clinical outcomes of insular gliomas.^10^, ^11^ Furthermore, Simon proved that the tumors of the limbic and paralimbic systems had a worse prognosis when the frontal lobe was involved.^12^ Subsequently, Berger et al. introduced a classification allowing for the preoperative prediction of insular glioma EOR.^13^ Other studies also described the relationship between tumor location and clinical features.^14^, ^15^ Previous studies inspired us deeply and we conceptualized our classification based on these.

Glioblastoma often has remarkable imaging features and is recommended to be maximum resected as early as possible. Its preoperative diagnosis and treatment strategy are relatively clear. However, the therapeutic strategies for nonglioblastoma remain the value of discussion. Evidence has shown that *IDH* wild‐type (*IDH*wt) glioma significantly differs in prognosis from other gliomas, strikingly resembling glioblastoma.^16^, ^17^ Achieving maximum tumor resection for this type of tumor can prolong patient survival. In contrast, appropriate tumor residue can reduce postoperative complications for tumors with low invasiveness and sensitivity to radiotherapy and chemotherapy. Considering the characteristics of the insula described above, preoperative prediction of tumor invasiveness of insular gliomas is of great significance in helping formulate treatment strategies. As well as the tumor size and spread pathway often reflect the invasiveness of the tumor. Accordingly, we proposed a tumor classification based on tumor spread.

In the present study, we reviewed a consecutive series of histological grade 2 and 3 insular glioma patients and established a nomogram model to predict patients' prognosis, meanwhile exploring the relationship between the proposed classification and clinical features.

## 2 METHODS

### 2.1 Patients

We retrospectively analyzed 283 patients who received the first surgical treatment at Beijing Tiantan Hospital from March 2011 to August 2021. Their surgery was performed by the same surgeon (JX), and mature electrophysiological techniques were performed to reduce the impairment of functions. The detailed surgical procedure can be found in our previous studies.^18^, ^19^ All patients were diagnosed with histological grade 2 and 3 insular gliomas based on clinical features, MRI imaging, and histopathological analysis. Those who received special preoperative treatment, such as radiotherapy and chemotherapy, were excluded. Tumors were pathologically examined after surgery and classified according to the 2016 World Health Organization (WHO) primary central nervous system (CNS) tumors. Informed consent was obtained from all participants in the study.

### 2.2 Tumor mapping and volume calculation

We used Mricron (https://www.mricro.com/) to perform manual tumor mapping and calculate tumor volume. Enhanced MRI T1 and MRI T2 sequences were used to evaluate the extent of tumors. All voxels with altered signal and adjacent edema were included in the regions of interest (ROIs). Two trained neurosurgeons identified tumor lesions with the guidance of an experienced radiologist. They were blinded to patients' characteristics, and a senior neurosurgeon decided the final ROI. The extent of resection (EOR) was calculated as follows: [(preoperative‐postoperative tumor volume)/preoperative tumor volume] × 100%. Tumor resections were classified into three types according to the EOR: gross total resection (GTR, EOR ≥ 90%), subtotal resection (90 > EOR ≥ 70%), and partial resection (EOR < 70%). Given the influence of postoperative edema and “residual triangle” proposed in our previous study, we did not group patients who achieved 100% resection.^19^

We also calculated the tumor growth rate (TGR) for patients with at least two preoperative MRIs with a minimum interval of 1 month. The calculation was performed using a previously proposed equation^20^, ^21^:

$$\mathrm{TGR}=100\times \left(\exp\;\left[\mathrm{TG}\right]-1\right).$$

$$\mathrm{TG}=\left(\mathrm{Ln}\left[V2/V1\right]/\text{time}\right).$$

where TG = tumor growth, V1 = tumor volume at date 1, V2 = tumor volume at date 2, and time (months) = ((date 2 – date 1 + 1)/30.44).

### 2.3 Tumor classification

In this study, we proposed a classification based on tumor spread. JX, BWX, ZYL, and ZGH conceptualized and designed the classification. BWX, ZYL, and ZGH classified the 283 tumors. They had more than 5–10 years of clinical experience and were blinded to patients' characteristics. A senior neurosurgeon, JX, and an experienced radiologist, SJS, provided consulting assistance. Tumors restricted to the paralimbic system were defined as type 1. When tumors simultaneously invade the limbic system (mainly referred to as the hippocampus and its surrounding structures in this study), they are defined as type 2. On this basis, tumors with additional internal capsule involvement were defined as type 3. Moreover, type 1 can be further classified as 1A and 1B according to whether it is purely insular glioma. Type 2 can be divided into 2A and 2B according to whether the hippocampus is involved. Type 3 can be divided into 3A and 3B according to whether the thalamus is involved.

### 2.4 Follow‐up

Patients underwent outpatient review within the first 3 months after surgery. Subsequently, we confirmed the patient's clinical status by telephone consultation or outpatient. When patients suffer from newly postoperative neurological deficits lasting more than 3 months, we will diagnose it as a long‐term complication.

### 2.5 Statistical analyses

SPSS V23.0 software (IBM Corp, Armonk, NY, USA) was used to perform statistical analyses. The chi‐square test and *t*‐test were performed for statistical comparisons of categorical and continuous variables. A nonparametric test was used when the continuous variables' distribution did not coincide with the normal distribution. Survival analysis was performed using the Kaplan–Meier approach with the Cox proportional hazards model. Overall survival (OS) was defined as the time from primary surgery to death, and progression‐free survival (PFS) was the time from primary surgery to recurrence of tumors judged by follow‐up MRI or death. *p* < 0.05 was considered statistically significant.

## 3 RESULTS

### 3.1 Demographics

The baseline characteristics of 283 patients with WHO grade 2–3 insular gliomas were summarized in Table 1. These patients included 154 males (54.4%) and 129 females (45.6%), with a median age of 40 years (range 18–70 years). The tumors grew on the left side in 143 patients (50.5%). The most common presenting chief complaint was seizures (156, 55.1%). When patients performed surgery, the median volume of the tumor was 70.7 cm^3^ (range 2.8–245.5 cm^3^). In this study, 199 patients (70.3%) underwent GTR, 66 patients (23.3%) underwent subtotal resection, and 18 patients (6.3%) underwent partial resection. Adjuvant therapy data were available for 259 patients, and of these patients, 41 patients (15.8%) received chemotherapy, 51 patients (19.7%) received radiotherapy, and 104 patients (40.2%) received radiotherapy plus chemotherapy, 63 patients (24.3%) received craniotomy alone.

**TABLE 1:** Characteristics of patients with insular glioma according to the classification.

Characteristic	Total	Classification			*p* value
		1	2	3	
Number of patients	283	140	63	80	
Age(years)					
≥40	153 (54.1%)	64 (45.7%)	33 (52.4%)	56 (70.0%)	**0.002**
Median	40	38	40	43	
Sex (Male)	154 (54.4%)	79 (56.4%)	34 (54.0%)	41 (51.3%)	0.757
Side (Left)	143 (50.5%)	72 (51.4%)	26 (41.3%)	45 (56.3%)	0.197
Preoperative seizure	156 (55.1%)	68 (48.6%)	34 (54.0%)	43 (53.8%)	0.674
Tumor volume (cm^3^)					
≥70	136 (48.1%)	36 (25.7%)	34 (54.0%)	66 (82.5%)	**<0.001**
Median	70.7	47.5	75.1	114.4	
Enhancement	93 (32.9%)	26 (18.6%)	21 (33.3%)	46 (57.5%)	**<0.001**
Histological grade 3	97 (34.3%)	27 (19.3%)	30 (47.6%)	40 (50.0%)	**<0.001**
1p/19q	232				
Codeletion	90 (38.8%)	48 (40.7%)	17 (36.2%)	25 (37.3%)	0.829
Intact	142 (61.2%)	70 (59.3%)	30 (63.8%)	42 (62.7%)	
NA	51				
MGMT	203				
Methylation	142 (70.0%)	75 (71.4%)	30 (71.4%)	37 (66.1%)	0.699
Unmethylated	61 (30.0%)	30 (28.8%)	12 (27.9%)	19 (33.9%)	
NA	80				
*IDH*1/2	177				
Mutation	138 (78.0%)	76 (87.4%)	34 (82.9%)	28 (57.1%)	**<0.001**
Wild‐type	39 (22.0%)	11 (12.8%)	7 (16.7%)	21 (42.9%)	
NA	106				
TERT	152				
Mutation	66 (43.4%)	35 (43.8%)	12 (37.5%)	19 (47.5%)	0.759
Wild‐type	86 (56.6%)	45 (56.3%)	20 (62.5%)	21 (52.5%)	
NA	131				
Ki‐67 index	165				
≥10%	88 (53.3%)	39 (44.3%)	19 (54.3%)	30 (71.4%)	**0.015**
<10%	77 (46.7%)	49 (55.7%)	16 (45.7%)	12 (28.6%)	
NA	118				
EOR					
≥90%	199 (70.3%)	115 (82.1%)	44 (69.8%)	40 (50.0%)	**<0.001**
<90%	84 (29.7%)	25 (17.9%)	19 (30.2%)	40 (50.0%)	
Additional treatment	259				
None	63 (24.3%)	40 (28.6%)	9 (14.3%)	14 (19.2%)	
Chemo	41 (15.8%)	17 (13.5%)	10 (16.7%)	14 (19.2%)	
Radiotherapy	51 (19.7%)	26 (20.6%)	16 (26.7%)	9 (12.3%)	
Radiotherapy and chemo	104 (40.2%)	44 (34.9%)	24 (40.0%)	36 (49.3%)	
NA	24				
Postoperative complication	243				
Motor impairment	19 (7.8%)	6 (4.9%)	4 (7.5%)	9 (13.2%)	0.123
Language disorder	17 (7.0%)	8 (6.6%)	4 (7.5%)	5 (7.4%)	0.964
NA	40				

*Note*: Boldface type indicates statistical significance.

Abbreviations: Chemo, chemotherapy; EOR, extent of resection; IDH, isocitrate dehydrogenase; MGMT, methylguanine methyltransferase gene; TERT, telomerase reverse transcriptase gene.

**FIGURE 1:**
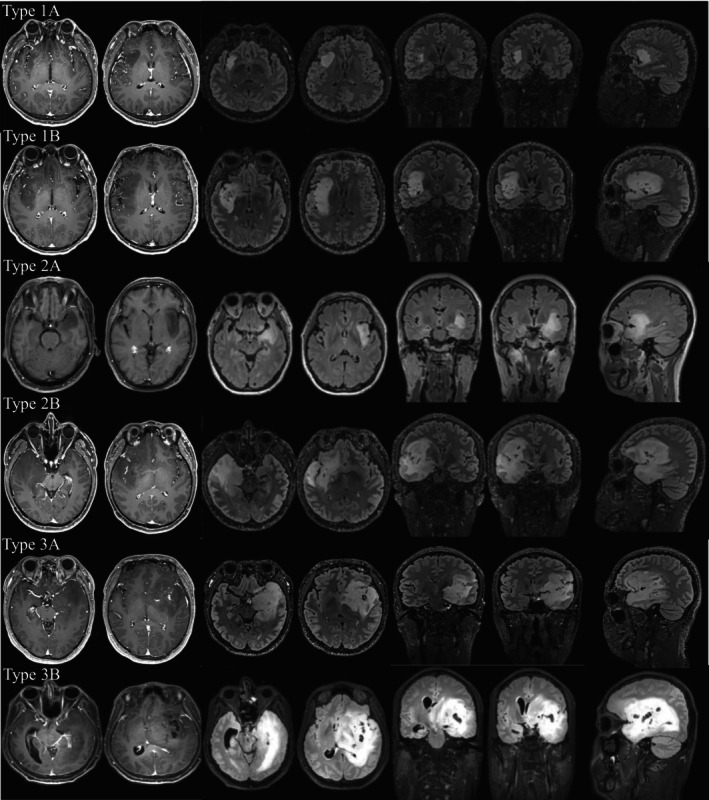
Schematic diagrams of the proposed classification. Every single row represents a demonstrative case. Type 1A represents a purely insular glioma, and type 1B is accompanied by an orbitofrontal cortex or temporal pole involved. Type 2A mainly invaded the structures surrounding the hippocampus such as the amygdala and parahippocampal gyrus while type 2B invaded the hippocampus. Type 3A invaded the internal capsule and type 3B had an additional thalamus involved.

Schematic diagrams of our classification are shown in Figure 1. According to our classification, the distribution of tumor spread was as follows: type 1A in 32 (11.3%) patients, type 1B in 111 (39.2%) patients, type 2A in 49 (17.3%) patients, type 2B in 11 (3.8%) patients, type 3A in 57 (20.1%) patients, type 3B in 23 (8.1%) patients. We also compared the characteristics of patients according to the complete proposed classification (Supplemental 1—Data S1). According to Table 1, there was a significant difference in preoperative tumor volume between different classifications (*p* < 0.001). Tumors defined as type 3 had a higher age at diagnosis (*p* = 0.002) and a higher preoperative volume (*p* < 0.001). In addition, the distribution of the enhanced tumors between our proposed tumor types was significantly different (*p* < 0.001). Furthermore, tumors defined as type 3 were more likely to be diagnosed as histological grade 3 (*p* < 0.001) and *IDH* wild type (*p* < 0.001), with a higher rate of Ki‐67 index (*p* = 0.015) and a lower rate of GTR (<0.001).

### 3.2 Histopathological and molecular characteristics of the tumors

Histopathological results confirmed 186 grade 2 glioma patients (65.7%) and 97 grade 3 glioma patients (34.3%). There was no apparent difference in both age (*p* = 0.146), sex (*p* = 0.341), and side (*p* = 0.454) between the two cohorts, but the Ki‐67 index differed obviously (median 5% vs. 20%; Mann–Whitney *U*‐test, *p* < 0.001). The volume of grade 3 tumors was more considerable than grade 2 tumors (median volume 108.7 cm^3^ vs. 48.2 cm^3^; Mann–Whitney *U*‐test, *p* < 0.001). Accordingly, GTR was more likely to be achieved in grade 2 gliomas than in grade 3 gliomas (145/186, 78.0% vs. 45/97, 46.4%; *p* < 0.001).

The molecular subtypes of tumors according to 2021 WHO classification are presented in Supplement 2—Data S1. There were 59 patients (34.9%) diagnosed as *IDH*mut with 1p/19q codeleted, 71 patients (42.0%) diagnosed as *IDH*mut with 1p/19q non‐codeleted, and 39 patients (23.1%) diagnosed as *IDH* wild type. There was no significant difference in age, sex, side, tumor volume, and the incidence of preoperative seizure between the three subtypes. Tumors defined as 1 were most likely diagnosed as *IDH*mut with 1p/19q non‐codeleted (38/79, 48.1%; *p* = 0.029). Tumors defined as type 3 had the highest rate of *IDH* wild type (21/45, 46.7%; *p* < 0.001). In the meantime, *IDH* wild type tumors were more challenging to achieve GTR (*p* = 0.009).

### 3.3 Tumor distribution and growth rate

The most frequent structures involved by insular gliomas were orbitofrontal cortex (180, 63.6%), temporal pole (179, 63.2%), amygdala (124, 43.8%), internal capsule (83, 29.3%), hippocampus (43, 15.1%), thalamus (23, 8.1%), corpus callosum (20, 7.0%), cingulate gyrus (17, 6.0%). Thus, we can see that insular tumors mainly extend to the paralimbic system and limbic system.

According to Berger‐Sanai classification, the distributions of tumors were revealed: zone I in 105 (37.1%) patients, zone II in 5 (1.7%) patients, zone III in 6 patients (2.1%), zone IV in 15 patients (5.3%), zone I + II in 34 patients (12.0%), zone I + IV in 63 patients (22.2%), zone II + III in 10 patients (3.5%), zone III + IV in 12 patients (4.2%), and giant in 33 patients (11.6%).

In the meantime, we analyzed data of patients with at least two preoperative MRI images (Table 2). We found that tumors defined as type 1 have a slower tumor growth rate than type 2 (mean 3.3%/month vs. 19.8%/month; Mann–Whitney *U*‐test, *p* < 0.001), so an early surgical intervention seemed more imperative. Typical images were shown in Supplement 3—Data S1. Nevertheless, there were two patients whose tumor classification transformed over 2 months. Accordingly, a repeat MRI examination is necessary to help us determine tumor classification.

**TABLE 2:** Data of patients with at least two preoperative MRI images.

Case	Earliest images		Latest images		Intervals (months)	TGR (%/month)	Histological grade	2021 WHO Molecular subtype	Ki‐67 index
	Tumor volume (cm^3^)	Tumor classification	Tumor volume (cm^3^)	Tumor classification					
1	9.3	1	9.5	1	6.2	0.3	2	3	NA
2^b^	40.8	1	44.6	1	17.8	0.5	3	2	NA
3	94.7	1	97.1	1	2.1	1.2	2	1	2%
4	89.0	1	92.8	1	3.6	1.2	2	NA	10%
5^b^	24.2	1	41.9	1	43.3	1.3	2	2	5%
6	35.2	1	59.2	1	33.2	1.5	2	NA	NA
7	53.7	1	63.7	1	7.5	2.3	2	NA	5%
8	51.4	1	78.1	1	17.2	2.5	2	NA	5%
9	12.9	1	29.7	1	28.3	3.0	2	1	5%
10	41.1	1	44.2	1	2.3	3.2	2	2	NA
11	76.3	1	79.9	1	1.4	3.3	2	1	10%
12	60.2	1	154.0	1	19.0	5.1	3	3	30%
13	21.0	1	22.4	1	1.1	6.0	2	2	3%
14	103.9	1	111.8	1	1.2	6.3	2	2	10%
15	3.1	1	46.6	1	1.2	7.2	2	2	NA
16	33.0	1	36.1	1	1.2	7.8	2	2	10%
17	84.6	2	93.7	2	1.0	10.8	3	NA	NA
18	117.8	2	165.0	2	2.6	13.8	3	NA	20%
19^a^	33.9	1	41.9	2	1.0	23.6	2	3	NA
20^a^	34.3	1	43.7	2	1.1	24.6	3	2	15%
21	89.5	2	115.8	2	1.1	26.4	2	2	6%
22^a^	29.7	2	116.4	3	38.6	3.6	3	NA	NA
23^a^	8.4	1	139.2	3	52.9	5.3	3	NA	NA
24	52.4	3	75.6	3	1.0	36.7	3	2	15%

*Note*: 2021 WHO molecular subtype: 1 = IDHmut with 1p/19q codeleted; 2 = IDHmut with 1p/19q non‐codeleted; 3 = IDH wild type.

Abbreviation: TGR, tumor growth rate.

^a^ The tumor type changed during the observation period.

^b^ The images of the two cases were shown in Figure 4.

### 3.4 Survival analysis

**FIGURE 2:**
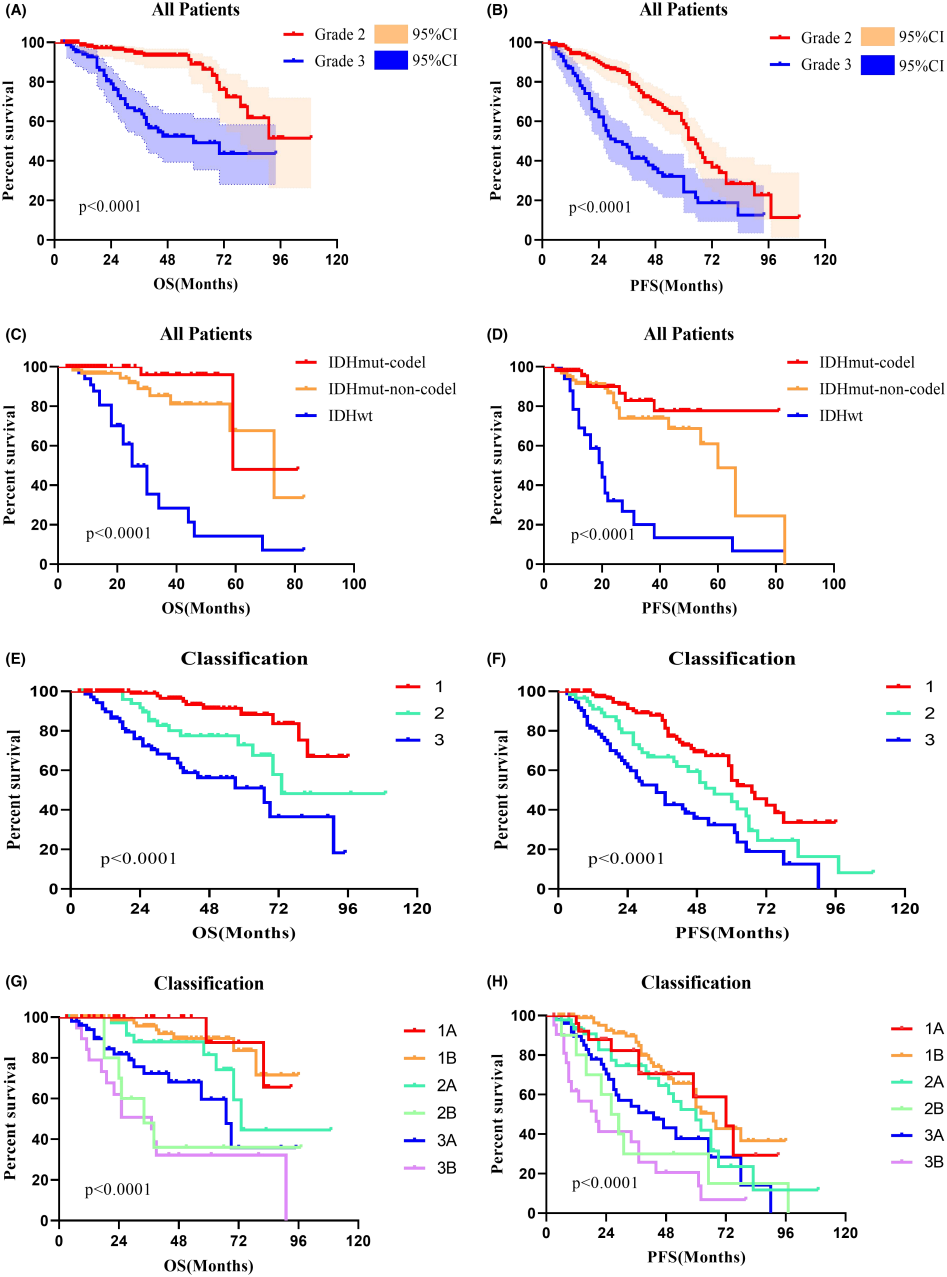
Kaplan–Meier survival curves show the *p* value of both overall survival (OS) and progression‐free survival (PFS) using log‐rank testing. (A, B) Histopathology has prognostic value in OS and PFS. (C, D) The 2021 WHO classification shows predictive value in both OS and PFS. (E–H) The prognostic value of the proposed classification.

**FIGURE 3:**
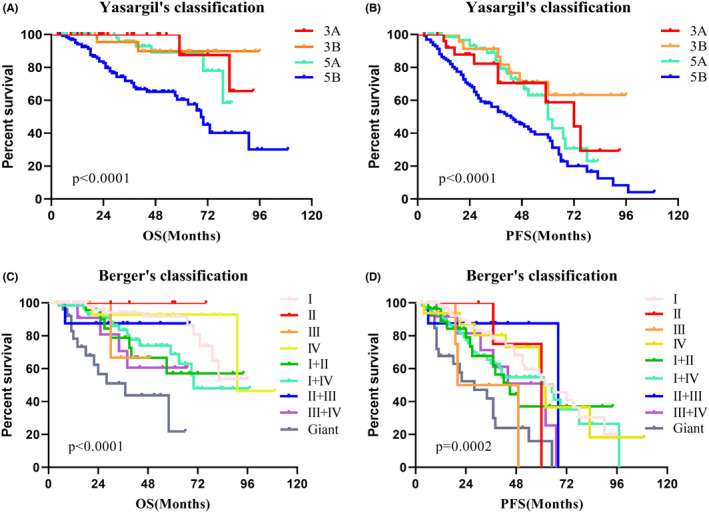
Kaplan–Meier survival curves of Yasargil's and Berger's classification. (A, B) The prognostic role of Yasargil's classification in OS and PFS. (C, D) The prognostic role of Berger's classification in OS and PFS.

Univariate survival analysis revealed that histological grade 2 patients had a better prognosis than histological grade 3 patients, both in OS (*p* < 0.0001; Figure 2B) and PFS (*p* < 0.0001; Figure 2B). The 2021 WHO molecular classification has predictive significance for both OS (*p* < 0.0001; Figure 2C) and PFS (*p* < 0.0001; Figure 2D). When comparing survival across tumor types, a lower OS (*p* < 0.0001; Figure 2E) and PFS (*p* < 0.0001; Figure 2F) were observed in type 3. Furthermore, there is a more significant difference in OS (*p* < 0.0001; Figure 2G) and PFS (*p* < 0.0001; Figure 2H) between type 3B and other types. We also compared the predictive role of both Yasargil and Berger classification in survival analysis (Figure 3). Although significant differences can be observed, some limitations are worth mentioning, and we will illustrate them in the discussion.

**FIGURE 4:**
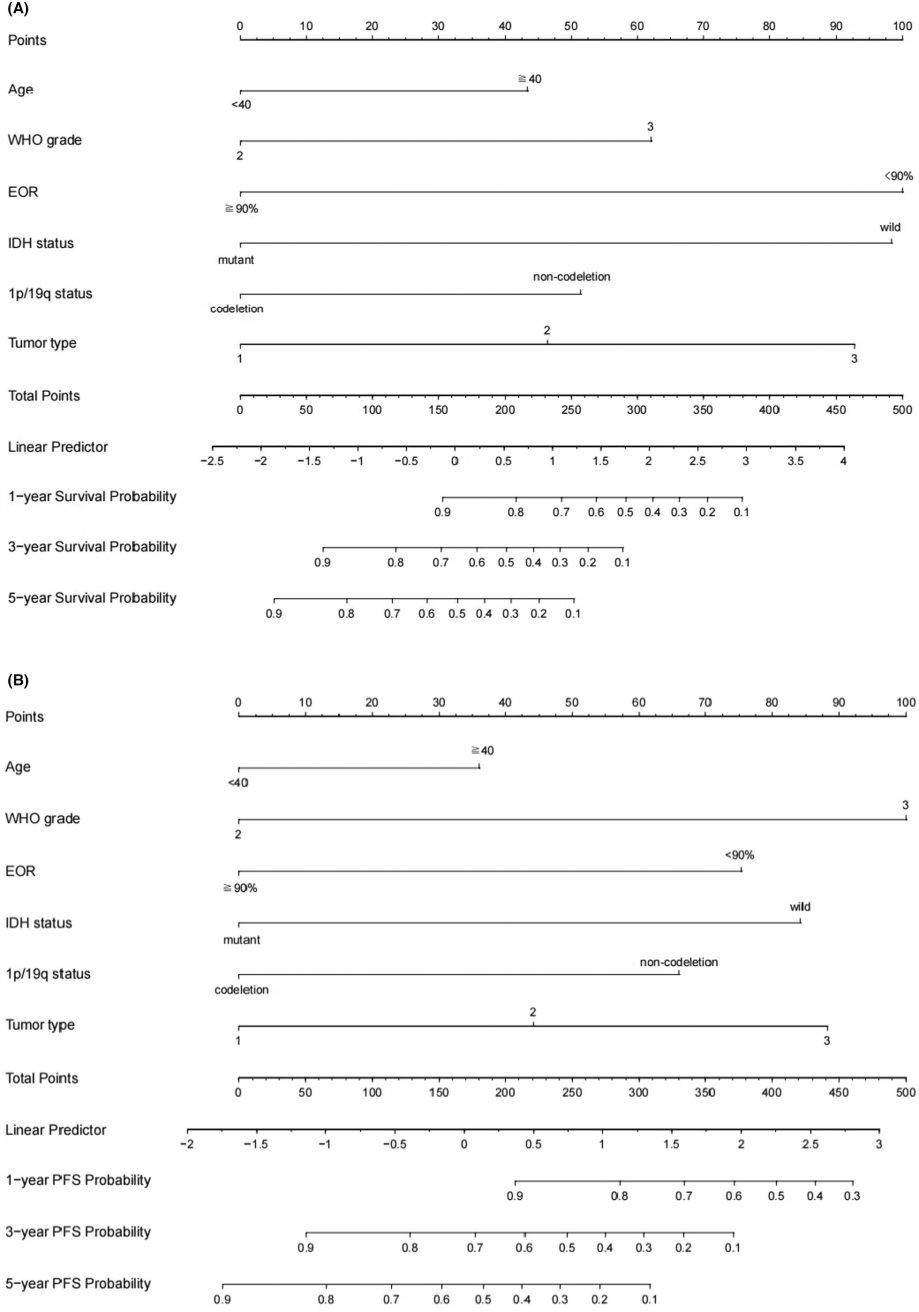
The individualized prediction models for OS and PFS in lower‐grade insular gliomas. (A) Prognostic nomogram to predict the 1, 3, and 5‐year survival probabilities. (B) Prognostic nomogram to predict the 1, 3, and 5‐year progression‐free survival probabilities.

Multivariate Cox regression analysis (variables: age, histological grade, the extent of resection, IDH status, 1p/19q status, tumor spread type) the extent of resection (HR 0.259, *p* = 0.004), IDH status (HR 3.694, *p* = 0.012), and tumor spread type (HR = 1.874, *p* = 0.031) as independent predictors of OS (Table 3). Histological grade (HR 2.609, *p* = 0.008), the extent of resection (HR 0.488, *p* = 0.038), IDH status (HR 2.225, *p* = 0.025), and tumor spread type (HR 1.531, *p* = 0.038) were significant in predicting PFS. We presented the results in the form of nomogram models (Figure 4).

**TABLE 3:** Multivariate analysis of survival outcomes.

Factor		OS			PFS		
		HR	95%CI	*p* Value	HR	95%CI	*p* Value
Age (years)	≥40	1.752	0.751–4.085	0.194	0.697	0.377–1.285	0.248
Histological grade	Grade 3	2.326	0.851–6.359	0.100	2.609	1.277–5.327	**0.008**
EOR (%)	≥90	0.259	0.103–0.652	**0.004**	0.488	0.247–0.962	**0.038**
IDH wild‐type	Yes	3.694	1.336–10.213	**0.012**	2.225	1.106–4.474	**0.025**
1p/19q codeletion	Yes	0.499	0.173–1.438	0.198	0.534	0.249–1.146	0.108
Tumor classification	3	1.874	1.058–3.320	**0.031**	1.531	1.023–2.292	**0.038**

*Note*: Boldface type indicates statistical significance.

Abbreviations: CI, confidence interval; HR, hazard ratio; OS, overall survival; PFS, progression‐free survival.

### 3.5 Postoperative new neurological deficit

Long‐term (>6 months) follow‐up data of complications were available for 243 (85.9%) patients. After surgery, new neurological deficits arose in 12.3% (30/243) of the patients. In this study, 7.8% (19/243) of the patients suffered from motor impairment, and 78.9% (15/19) patients made a functional recovery. Language impairment occurred in 7.0% (17/243) of the patients, and 88.2% (15/17) of the patients completely or nearly completely recovered. 2.9% (7/243) of the patients developed both motor and language disorders. Tumors defined as type 3 had a slightly higher risk of postoperative motor impairment than other types, but there is no significant difference (*p* = 0.123). One important reason is that many patients exhibit movement disorders before surgery.

## 4 DISCUSSION

In this research, we proposed a classification based on the anatomical relationship and spread pathway of insular gliomas. Since glioblastoma has distinctive imaging features and clear surgical indications, and the abnormal signal band around the tumor is difficult to identify as tumor invasion or angiogenic edema, we only classified insular gliomas diagnosed as histological grades 2 and 3 in this study. However, there are also significant differences in the properties between histological grades 2 and 3 tumors. It is tough to distinguish them through preoperative images. Our study revealed there was a considerable rate of grade 2 tumors with preoperative enhancement (42/186, 22.5%) and grade 3 tumors without preoperative enhancement (51/97, 52.5%). A multidimensional evaluation is necessary to improve the accuracy of preoperative diagnosis. We hold that different tumor spread type represents different tumor invasiveness, which is helpful to further differentiate them.

Due to its infiltrative nature and particular position, insular gliomas have a unique spread pathway. Proximity to surrounding structures and fasciculi facilitates tumor diffusion.^13^ Also, previous studies mentioned that the vascularization pattern to the limbic and paralimbic systems is unique, bearing possible importance in the spread of tumors.^22^, ^23^, ^24^, ^25^ Yasargil put forward the classification accordingly, initiating the attention of experts to the diffusion pattern of insular tumors.^5^, ^11^, ^26^, ^27^ The classification inspired us deeply, and we improved and proposed our classification accordingly. We considered purely insular gliomas to be the early stage and named them type 1A. The uncinate fasciculus interconnects the paralimbic system. Insular tumors tended to spread to one or two other structures of the paralimbic system, and we named them type 1B. Tumors defined as type 1 tend to be confined in the paralimbic system and have a slow tumor growth rate. Their nature and biological characteristics (specific manifestations include molecular pathology, prognosis, and tumor growth rate) are relatively better than other types. Previous research also showed that the insula is strongly connected to the thalamus and structures of the limbic system, such as the amygdala and hippocampus.^28^, ^29^ We accordingly refined the classification as type 2 and type 3. Other structures of the limbic system such as the cingulate gyrus were rarely invaded and not suitable as a criterion for type evaluation. To help readers identify relevant structures and understand our classification, we provide an additional diagram with labels (Supplemental 4—Data S1).

The nature and prognosis of type 2 tumors are between the type 1 and type 3. Some researchers hold that hippocampal involvement independently predicts worse survival.^26^ In this study, a significantly shorter OS was also observed in tumors involving the hippocampus (median 44 months, *p* < 0.0001) than in tumors without hippocampus involvement (median not reached, *p* < 0.0001). Thus, we divided type 2 tumors into 2A and 2B according to whether they invade the hippocampus. Compared with other classifications, tumors defined as type 3 seemed more aggressive than other types. Resection for thalamic tumor is usually challenging, and insular gliomas with thalamus involvement had significantly shorter OS (median 37 months, *p* < 0.0001) than tumors without thalamus involvement (median not reached, *p* < 0.0001). Accordingly, type 3 tumor was divided into 3A and 3B according to whether it invaded the thalamus. Although tumors defined as type 3B were challenging to resect thoroughly and had a poor prognosis, they tended to decrease in recent years with the popularization of the general health examination. More and more tumors are detected in the early stages.

The research proposed by Professor Yasargil, including Professor Zentner, mainly focuses on the paralimbic and limbic systems.^5^, ^9^ We performed survival analysis for their classification and observed significant differences (Figure 3A,B). However, there were some notable issues. Firstly, the prognosis of 5 B‐type tumors is significantly poor, while the prognosis of the other three types is similar. It indicates we can further refine the classification. In addition, many insular gliomas invaded the basal ganglia, but previous classifications ignored these tumors. Yasargil's classification of 5B tumors covers type 2 and 3 tumors of our classification, but we believe there are diversities between the tumors. Therefore, we conducted a survival analysis between the type 2 and 3 tumors and found significant differences in both OS (*p* = 0.030) and PFS (*p* = 0.036). The putamen is a good evaluation indicator to judge basal ganglia invasion, but tumors involved in the basal ganglia tend to be huge. The putamen is often compressed intensely and is difficult to distinguish. Hence, we chose the internal capsule as the indicator of whether the tumors can be defined as type 3. The invaded internal capsule can show an obvious high signal on T2 or flair image, which is relatively easy to identify. However, the number of type 2B tumors is relatively low and makes the corresponding results instable, which can be seen in Figure 2. We will revalidate the results by including more cases in future research.

Berger‐Sanai classification referred to the Sylvian fissure and the foramen of Monro and divided Zones into I, II, III, and IV. It has strong clinical practicability, and our previous study has verified that it is helpful for surgical strategies.^18^ However, this classification is mainly based on the tumor location and is not good at evaluating the specific extent of tumor invasion. For example, a tumor defined as zone I can be a pure insular glioma or a huge tumor invading both orbitofrontal and basal ganglia. The two types of tumors may be completely distinct. We also analyzed the prognostic role of the Berger‐Sanai classification (Figure 3C,D) and learned that the prognosis of the giant tumor was poor (median OS 37 months, *p* < 0.0001). However, the giant tumor only accounts for a small part, and the prognosis of other tumors is irregular, which makes it difficult to achieve a comprehensive summary.

Besides the known connection between the insula and its surrounding structures, some phenomena found in clinical practice also facilitated our classification. Many tumors are confined to the paralimbic system for a long time. They tended to stay in the region between the temporal pole and the anterior part of the uncus. Some tumors could continue to affect the limbic system with a small volume, while others failed to squeeze into it even if they had a considerable size. The differences in tumor properties may contribute to that. The region mentioned above may be a transition region between the paralimbic and limbic systems. Most type 1 (107/140, 76.4%) and type 2 (62/63, 98.4%) tumors invaded this region, and the transition region may be one of the spread pathways of the insular gliomas. It was worth mentioning that although three cases invaded the internal capsule without affecting the limbic system, they had a small size and did not reach the transition region. This means these tumors were discovered in an early stage and may have just begun to grow. If the tumors continued to grow, the limbic system would also be invaded. For tumors defined as type 1, including the transition region into the scope of excision may be beneficial for preventing tumor spread, even though their transition regions are not invaded in preoperative images. Meanwhile, due to their relatively benign properties, appropriate tumor residues can be considered if we encounter intractable parts to remove. For tumors defined as type 2, they have already invaded the limbic system, so it can be considered to completely remove the hippocampus and its corresponding structures when we performed surgery. For tumors defined as type 3, their pathological results and prognosis could be poor, so a maximum resection except the basal ganglia is necessary.

In the present study, an incidental insular glioma was referred to as a glioma found in imaging examinations for a reason unrelated to the tumor. Incidental insular glioma accounts for 45.7% (64/140) of type 1 tumors. Considering the risk of postoperative complications and many other reasons, quite a lot of patients choose a temporary observation despite we recommend early surgery. When mentioning postoperative complications, we use the description of “nearly completely recovered,” which means that the patient's daily life and function have been restored. However, it still changes the patient's life quality to varying degrees, which can be a disaster for patients with special requirements. Perhaps they can maintain a normal life for a longer time and accomplish some major life events before surgical treatment. However, all they can get is “early surgery,” no matter where they consult. No one can help them evaluate the safety of the waiting period. We attempted to solve the dilemma by our classification. Type 1 tumors seemed to have a slower growth rate and a confined growth pattern. We can see it from Supplement 3—Data S1 and our clinical practice. Accordingly, appropriate delayed surgery and close observation may be available for some patients with tumors defined as type 1. Even if it develops into type 2A during the period under observation, its prognosis and extent of resection are still optimistic. However, if a diagnosis of type 2A is confirmed, surgery should be performed immediately and thoroughly to prevent it from developing into type 2B and type 3. Notably, our purpose is not to recommend patients give up early surgery, but to provide a possible research direction for evaluating the safety of the waiting period for patients who have to consider delaying the operation. As for type 3, our data showed some of them have a relatively slow growth rate, but this may be attributed to their large volume and insufficient space for their growth. Its resection should be performed as soon as possible owing to their properties. Absolutely, the reliability of the considerations about resection range and operation time needs to be further verified.

In the end, there are some limitations in our study. The molecular pathology status of many patients was not tested, especially for newly recognized glioblastoma markers. Its role in predicting prognosis was not completely confirmed. Moreover, it is somewhat challenging to identify the structures around the hippocampus, which sometimes were squeezed by the swollen insula and tumor. In addition, there is subjectivity in the evaluation of tumor types. Therefore, we reviewed multidimensional images to reduce the possibility of misjudgment. A more precise preoperative prediction of tumors may be achieved by combining existing findings and artificial intelligence.

## 5 CONCLUSION

The current study proposes a classification of the insular glioma according to the tumor spread. It indicates that the tumors defined as type 1 have a relatively better nature and biological characteristics, and those defined as type 3 can be more aggressive and refractory. Besides its predictive value for prognosis, the classification has potential value in formulating surgical strategies for patients with insular gliomas.

## AUTHOR CONTRIBUTIONS

**Bowen Xue:** Conceptualization (lead); data curation (lead); formal analysis (lead); investigation (lead); methodology (lead); software (lead); visualization (equal); writing – original draft (lead). **Zonggang Hou:** Conceptualization (supporting); investigation (supporting); methodology (supporting); project administration (supporting); supervision (supporting); visualization (equal); writing – review and editing (supporting). **Zhenghai Deng:** Investigation (supporting); methodology (supporting); supervision (supporting); validation (supporting). **Shengjun Sun:** Investigation (supporting); methodology (supporting); validation (supporting). **Chuanhao Zhang:** Data curation (supporting); formal analysis (supporting); investigation (supporting). **Yuesong Pan:** Formal analysis (supporting); methodology (supporting). **Yazhuo Zhang:** Investigation (supporting); supervision (supporting). **Zhenye Li:** Conceptualization (lead); methodology (supporting); supervision (supporting); validation (supporting); writing – review and editing (lead). **Jian Xie:** Conceptualization (lead); funding acquisition (lead); investigation (supporting); project administration (lead); supervision (lead); validation (lead); writing – review and editing (lead).

## FUNDING INFORMATION

This study was supported by the National Natural Science Foundation of China (ID: 82172028).

## CONFLICT OF INTEREST STATEMENT

The authors declare that they have no relevant financial or non‐financial interests to disclose.

## ETHICS STATEMENT

The studies involving human participants were approved by the Ethics Committee of Beijing Tiantan Hospital. And all methods were performed in accordance with the relevant guidelines and regulations.

## PATIENT CONSENT STATEMENT

Informed consent was obtained from all participants in the study.

## Supporting information


Data S1.

## Data Availability

The datasets used and/or analyzed during the current study are available from the corresponding author on reasonable request.
