# Supplementary material for: Survival outcome and predictors of WHO grade 2 and 3 insular gliomas: A classification based on the tumor spread
Source: Cancer Med. 2024 Jun 8;13(11):e7377. doi: 10.1002/cam4.7377 (PMC11161818; doi:10.1002/cam4.7377)
Supplement: Supplementary file 1 — Data S1. [file CAM4-13-e7377-s001.pdf]

Survival outcome and predictors of WHO grade 2 and 3 insular gliomas: A classification based on the tumor spread

Bowen Xue, MD,[1,2] Zonggang Hou, MD,[1,2] Zhenghai Deng, MD,[1,2] Shengjun Sun, MD,[2,3] Chuanhao Zhang, MD,[1,2] Yuesong Pan, MD,[2] Yazhuo Zhang, MD,[2,4] Zhenye Li, MD,[1,2*] and Jian Xie, MD[1,2*]

Journal name: Cancer Medicine

Correspondence:
Zhenye Li: Beijing Tiantan Hospital, Capital Medical University, China. Email: drlizhenye@163.com
Jian Xie: Beijing Tiantan Hospital, Capital Medical University, China. Email: xiejian0630@126.com

Supplemental 1. Characteristics of patients according to the complete classification

		Classification						
**Characteristic**	**All**	**1A**	1B	**2A**	**2B**	**3A**	**3B**	**P value**
Number of patients	283	32(11.3%)	111(39.2%)	49(17.3%)	11(3.8%)	57(20.1%)	23(8.1%)	
Age ≥40 years	153(54.1%)	14(43.8%)	51(45.9%)	26(53.1%)	6(54.5%)	38(66.7%)	18(78.2%)	**0.02**
Sex(Male)	154(54.4%)	18(56.3%)	61(55.0%)	28(57.1%)	6(54.5%)	26(45.6%)	15(65.2%)	0.690
Side(Left)	143(50.5%)	12(37.5%)	61(55.0%)	21(42.9%)	4(36.4%)	31(54.4%)	14(60.9%)	0.269
Tumor volume ≥70 cm^3	136(48.1%)	3(9.4%)	33(29.7%)	27(55.1%)	7(63.6%)	44(77.2%)	22(95.7%)	**<0.001**
Preoperative seizure	145(51.2%)	16(50.0%)	53(47.7%)	26(53.1%)	7(63.6%)	30(52.6%)	13(59.1%)	0.901
Tumor grade 3	97(34.3%)	4(12.5%)	23(20.7%)	22(44.9%)	8(72.7%)	25(43.9%)	15(65.2%)	**<0.001**
Molecular subtype	169(59.7%)	22	64	30	7	35	11	**<0.001**
1	59(34.9%)	9(40.9%)	25(39.1%)	13(43.3%)	0	11(31.4%)	1(9.1%)	
2	71(42.0%)	11(50.0%)	30(46.9%)	15(50.0%)	2(28.6%)	12(34.3%)	1(9.1%)	
3	39(23.1%)	2(9.1%)	9(14.1%)	2(6.7%)	5(71.4%)	12(34.3%)	9(81.8%)	
EOR <90%	84(29.7%)	7(21.9%)	18(16.2%)	11(22.4%)	8(72.7%)	26(45.6%)	14(60.9%)	**<0.001**

Molecular subtype (according to 2021 WHO CNS classification): 1 = IDHmut with 1p/19q codeleted; 2 = IDHmut with 1p/19q non-codeleted; 3 = IDH wild type. Boldface type indicates statistical significance.

Supplemental 2. Relationship between the clinical features and 2021 WHO molecular subtypes

		Molecular subtypes			
		***IDH*mut with 1p/19q codeleted**	***IDH*mut with 1p/19q non-codeleted**	***IDH* wild type**	**P value**
Total	169	59	71	39	
Age(≥40years)	89(52.7%)	35(59.3%)	30(42.3%)	24(61.5%)	0.068
Sex(Male)	85(50.3%)	25(42.4%)	39(54.9%)	21(53.8%)	0.319
Side(Left)	85(50.3%)	32(54.2%)	37(52.1%)	16(41.0%)	0.406
Tumor volume(≥70cm^3)	76(45.0%)	27(45.8%)	27(38.0%)	22(56.4%)	0.177
Preoperative seizure	79(46.7%)	29(49.2%)	28(39.4%)	22(56.4%)	0.210
Classification					
1	79(46.7%)	30(38.0%)	38(48.1%)	11(13.9%)	**0.029**
2	45(26.6%)	18(40.0%)	20(44.4%)	7(15.6%)	0.360
3	45(26.6%)	11(24.4%)	13(28.9%)	21(46.7%)	**<0.001**
Histological grade 3	61(36.1%)	19(32.2%)	21(29.6%)	21(53.8%)	**0.030**
Ki-67 index(≥10%)	72(42.6%)	30(50.8%)	22(31.0%)	20(51.3%)	0.056
EOR≥90%	120(71.0%)	45(76.3%)	56(78.9%)	19(48.7%)	**0.002**
Median OS(months)		NA	73	34	**<0.0001**
Median PFS(months)		NA	66	21	**<0.0001**

Mut= mutation; OS = overall survival; PFS = progression-free survival.
Boldface type indicates statistical significance.

Supplemental 3. Typical preoperative MRI images of two cases.

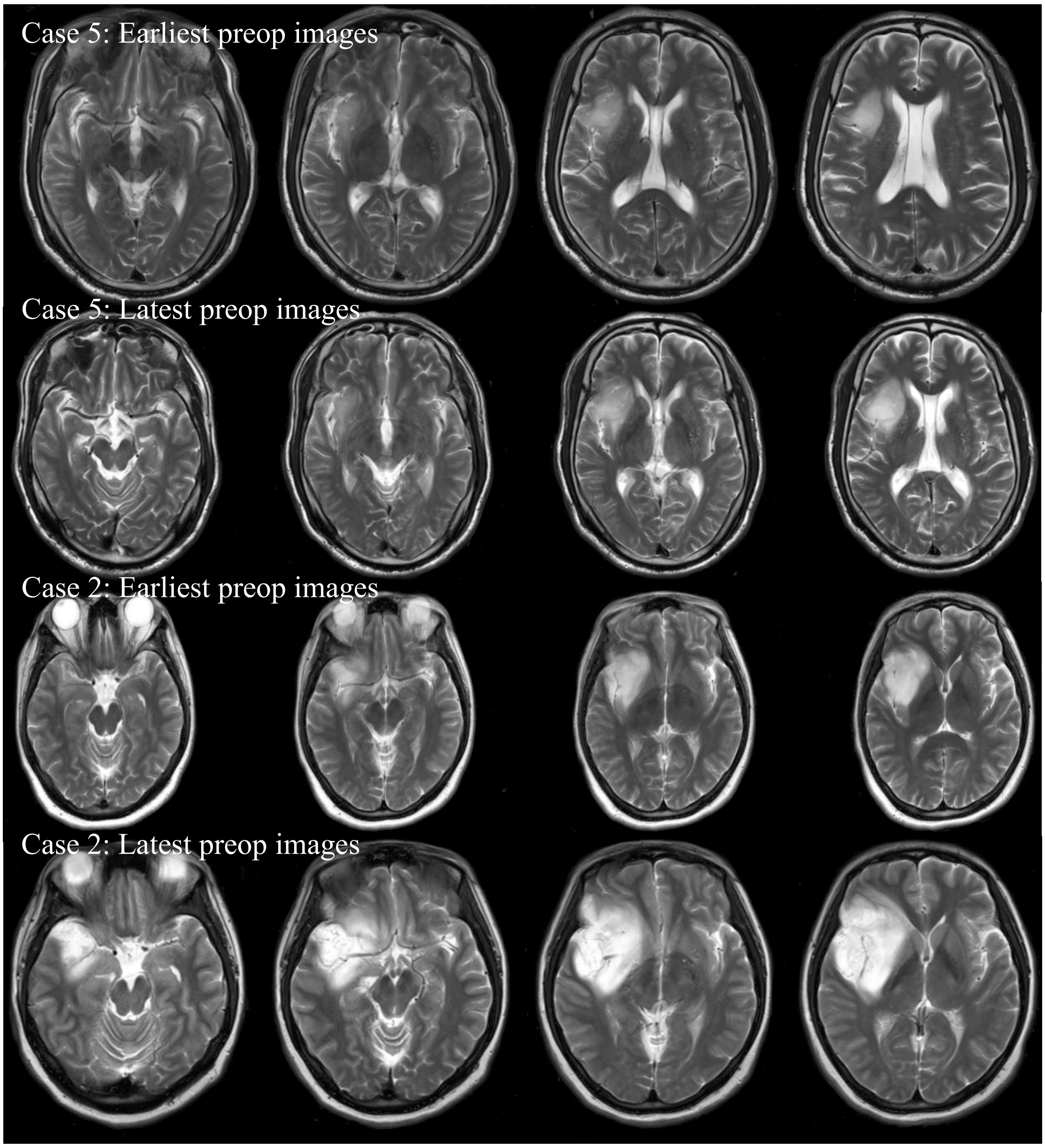

Case 5: tumor spread type 1A; examination intervals 43.3 months; TGR 1.3%/month.
Case 2: tumor spread type 1B; examination intervals 17.8 months; TGR 0.5%/month.

Supplemental 4. The schematic diagram for the tumor spread pathway

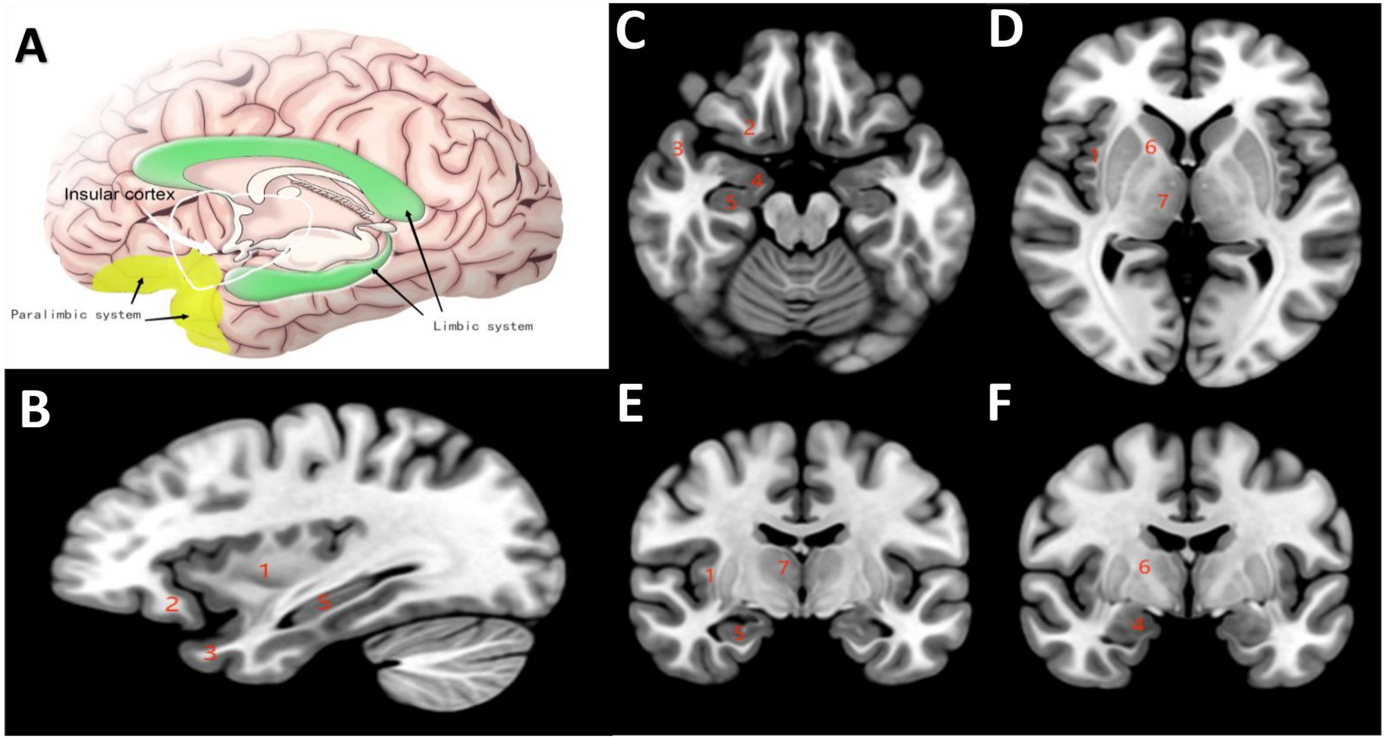

Panel A shows the position schematic diagram of the paralimbic system (including the insular cortex) and limbic system, and the connection between the two systems is the main diffusion pathway of insular gliomas. Panel B-F shows the most commonly invaded structures of insular gliomas in different dimensions, and they were labeled with numbers in the figure. 1-3 belong to the paralimbic system and tumors invading them are defined as type 1; 4-5 belong to the limbic system and tumors invading them are defined as type 2; tumors invading 6 and 7 are defined as type 3. Each type of tumor will be further divided into two groups (A and B), based on their invasion in various systems. The following is the structure names corresponding to the numbers: 1 Insula; 2 Orbitofrontal cortex; 3 Temporal pole; 4 Amygdala and parahippocampal gyrus; 5 Hippocampus; 6 Internal capsule; 7 Thalamus
